# A Unique Case of Multicentric Infantile Myofibromatosis with Radiologic-Pathologic Correlation

**DOI:** 10.3390/children9010053

**Published:** 2022-01-03

**Authors:** Luka Pušnik, Daja Šekoranja, Domen Plut

**Affiliations:** 1 Faculty of Medicine, University of Ljubljana, 1000 Ljubljana, Slovenia; plut.domen@gmail.com; 2 Institute of Pathology, Faculty of Medicine, University of Ljubljana, 1000 Ljubljana, Slovenia; daja.sekoranja@mf.uni-lj.si; 3 Clinical Radiology Institute, University Medical Centre Ljubljana, 1000 Ljubljana, Slovenia

**Keywords:** myofibromatosis, neonate, lung, ultrasonography, X-ray, immunohistochemistry

## Abstract

Infantile myofibromatosis (IM) is a rare condition with a variable clinical presentation that characteristically affects young children. Most frequently it presents as one or more benign nodules of the skin, bones, soft tissues, or, rarely, visceral organs. According to the location and number of lesions, there are three different forms: solitary, multicentric without visceral involvement, and multicentric with visceral involvement (generalised), with the latter having the least favourable prognosis. We present a unique case of severe congenital generalised IM in a new-born male who required intubation and mechanical ventilation immediately after the birth due to respiratory distress. A chest radiograph showed numerous tumours involving the entire lung, resembling a metastatic lung disease. Additionally, the neonate had multiple, bluish, papular skin nodules and a biopsy of a skin nodule ultimately led to the diagnosis of IM. Diffuse lung involvement prevented adequate ventilation which resulted in multiorgan failure and death before targeted treatment could have been initiated. The presented case is unique, as such atypical extensive involvement of the lung and leptomeninges in IM has not been reported before. In this brief report, we present the findings of radiographic and ultrasonographic examinations in correlation with autopsy and histopathology.

## 1. Introduction

Infantile myofibromatosis (IM) is a condition, sometimes congenital, characterised by the formation of one or more benign myofibromatous proliferations that affect the skin, bone, muscle, or internal organs. Although considered rare, IM is the most common benign fibrous tumour proliferation in infants [1]. Hitherto three different subtypes have been described in the literature: solitary, multicentric without visceral involvement, and generalised (i.e., multicentric with visceral involvement) [2]. IM usually manifests shortly after birth or in early infancy as a firm, flesh-coloured to purple nodule or multiple nodules, located in the skin or subcutaneous tissue. Most commonly it presents as a solitary cutaneous nodule or as several cutaneous and/or soft tissue nodules, that may range in size from a few millimetres to several centimetres and up to a few hundred may occur [3,4]. In both aforementioned IM subtypes, the nodules normally regress spontaneously within the first two years of diagnosis and generally do not metastasize [3,5]. Our case report represents the third and the rarest subtype of IM—a generalised form with visceral organ involvement where the nodules can affect every internal organ, including the central nervous system [1,6]. In contrast to the other two subtypes, this form of IM has a much worse prognosis with a mortality rate of up to 76% [3]. Death usually occurs due to gastrointestinal or cardiopulmonary compromise [6].

Herein, we present a unique case of generalised IM with visceral and leptomeningeal involvement, including multiple round nodules involving the entire lung, resembling a metastatic disease on chest radiography.

## 2. Case Presentation

**Figure 1:**
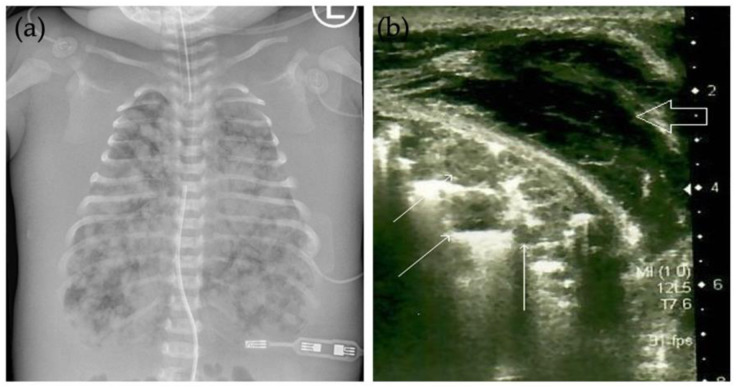
Frontal radiograph and ultrasonography of the lung. (**a**) Frontal chest radiograph shows multiple round lesions of different sizes diffusely involving the entire lung. Generalised body oedema in keeping with hydrops fetalis can be seen. Also noted are endotracheal tube, nasogastric tube, and umbilical venous line. (**b**) Ultrasonography of the lung showed multiple round hypoechoic solid nodules within the lung parenchyma (thin white arrows). No pleural effusion was seen. Severe body oedema is also noted (thick void arrow).

The patient was a full-term new-born male (4110 g) with hydrops fetalis, delivered by emergency caesarean section due to the pelvic insertion, polyhydramnios, ascites, and meconium in the amniotic fluid. The mother’s past medical and obstetric history included an uneventful pregnancy with a healthy female and an abortion at 6 weeks’ gestation. The Apgar scores of the neonate in the first, fifth, and fifteenth minutes were 1, 2, and 4, respectively. Immediately after birth, he required intubation and mechanical ventilation. The patient was bradycardic (85 bpm) and hemodynamically unstable; therefore, vasoactive support, along with a surfactant and antibiotics (ampicillin, gentamicin), was introduced in the intensive care unit. A chest radiograph disclosed multiple round lesions of various sizes diffusely involving the entire lung, which are extremely rare in neonates (Figure 1a). Their diameter was up to 8 mm. It was not possible to determine whether these were fluid or soft tissue changes by radiography; hence, ultrasonography of the lung was performed which showed that the multiple round nodules within the lung parenchyma were solid in nature (Figure 1b). There was no pleural effusion. Ultrasonography of the head discovered mild cerebral oedema, indicating mild hypoxic-ischemic encephalopathy, and ultrasonography of the abdomen was undertaken for assessment of the abdominal organs, which appeared normal. Biochemical analysis revealed elevated levels of liver enzymes including aspartate transaminase and gamma-glutamyl transferase, disturbances of blood coagulation tests with elevated D-dimer, and decreased thrombocyte levels. The neonate also had metabolic acidosis (pH = 7.11) with increased blood lactate concentrations, hence bicarbonate was introduced. The aforementioned coagulopathy was corrected and, due to anuria and the unclear aetiology of the disease, peritoneal dialysis was initiated.

**Figure 2:**
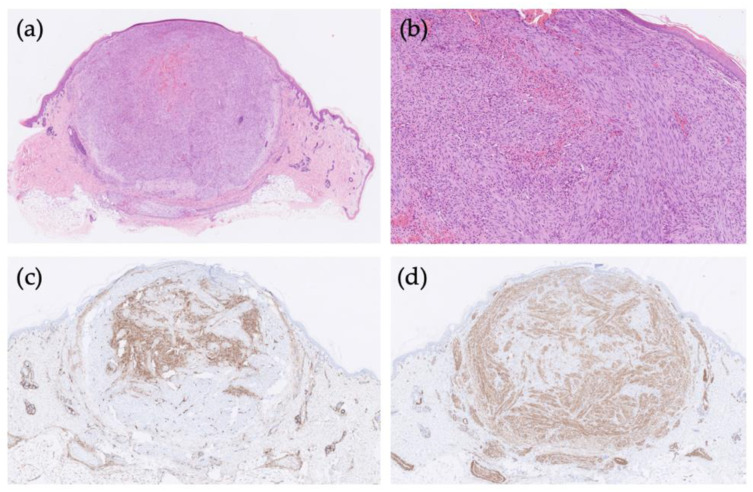
Histologic features of infantile myofibroma. The tumour shows a biphasic growth pattern with short fascicles of spindle cells surrounding centrally located round cells (hematoxylin-eosin [HE], (**a**) ×10, (**b**) ×100). Immunohistochemically, (**c**) round cells are positive for CD34, while (**d**) spindle myoid cells express smooth muscle actin (SMA).

Special attention was paid to the skin changes. The trunk and limbs of the new-born were covered with multiple, bluish, papular skin nodules, which averaged 4 mm in size. One of the skin nodules was biopsied and sent for histopathological assessment. The biopsy showed a well-demarcated biphasic tumour proliferation in the dermis, consistent with a myofibroma. One component represented short fascicles of bland, mature spindle myoid cells especially at the periphery of the tumour, while the other component consisted of smaller, more immature-looking round cells arranged irregularly around thin-walled staghorn-like vessels in the central portion of the tumour nodule (Figure 2a,b). Immunohistochemically, mature spindle cells were positive for smooth muscle actin and negative for CD34, while immature-looking round cells showed the opposite immunophenotype, being positive for CD34, but negative for smooth muscle actin (Figure 2c,d). Both cell types were negative for desmin and S100. Since the US appearance of the skin and lung lesions was similar, a diagnosis of generalised IM was settled upon.

Two days after the diagnosis, the new-born died due to cardiopulmonary failure. An autopsy was performed which revealed multiple myofibromatous tumour proliferations in the lungs, myocardium, skin, lymph nodes, leptomeninges, muscles, and intestine. All tumours showed similar histologic features to those in the skin biopsy, with variable proportions between the spindle and round cell components.

## 3. Discussion

The new-born in our case presented with a severe condition and various clinical symptoms. The most prominent changes were observed on the skin and lung. Radiographically, multiple round lesions of different sizes involving the entire lung were seen, which resembled a metastatic disease. This is an extremely rare finding in a new-born. A neuroblastic tumour (neuroblastoma or ganglioneuroblastoma) was first considered, as such cases have been reported in the literature [7]. No primary tumour was found by ultrasonography of the thorax and abdomen. Other metastatic diseases (child’s, placental, or maternal), as well as a diverse spectrum of infectious, autoimmune, metabolic, and hematologic diseases, were also considered [8]. Low inflammatory markers with a negative streptococcus test and negative tests for TORCH infections, including cytomegalovirus, herpes simplex virus, enterovirus, and parvovirus B19, reduced the possibility of an infection. Blood was negative for leukemic cells, ascites negative for malignant cells, and placental examination was normal. The US of the lung, however, revealed that the nodules were solid in nature. Additionally, in their sonographic appearance, the lung nodules were similar to the skin lesions. Computed tomography (CT) or magnetic resonance imaging (MRI) could have been used to demonstrate the appearance and extent of lung lesions with greater detail; however, they were not performed in our case due to the patient’s poor clinical condition and the lack of expected additional clinical value of CT or MRI, as they are not specific in the evaluation of solid lung nodules [9]. Biopsy of the skin lesions was performed and diagnosis of multicentric IM with visceral involvement was reached by histopathological evaluation.

**Figure 3:**
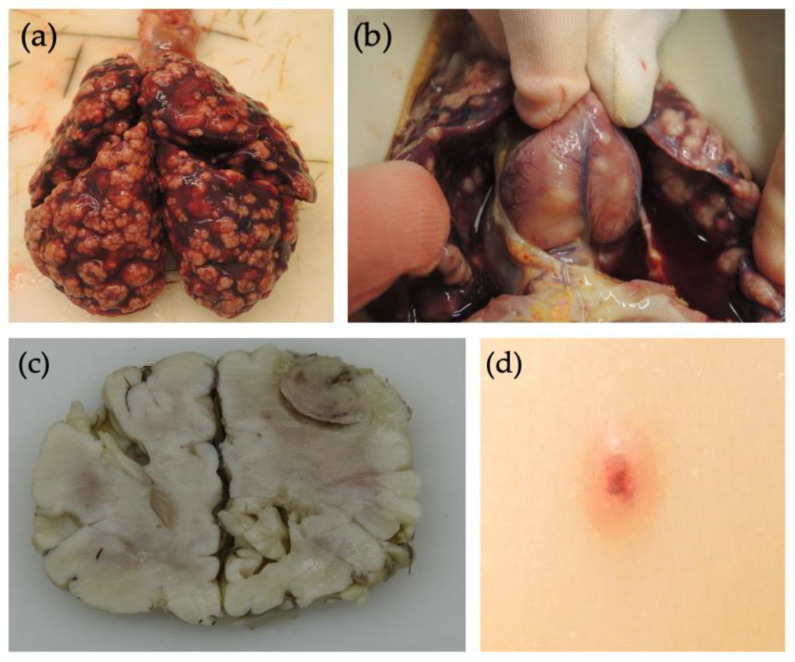
Autopsy of the neonate revealed numerous nodules in the (**a**) lung parenchyma, (**b**) myocardium, and (**c**) leptomeninges. (**d**) Bluish, papular nodule on the trunk of neonate representing myofibroma affecting the skin.

In 1954, the first description of IM was made by Stout and several case reports have been published since then [10]. Apart from recurrent mutations in PDGFRβ and NOTCH3 genes reported in some familial cases and sporadic tumours, the aetiology is often unknown [5,11]. The clinical appearance of myofibroma can be heterogeneous and vary in size, ulceration, and lobulation, hence biopsy is crucial for the diagnosis. As aforementioned, a small proportion (only about a quarter of cases) are multicentric, and only a third of these have visceral organ involvement, most frequently in the gastrointestinal tract [4]. In our case, the tumour nodules were small in size, up to 1 cm in diameter, affecting the skin, lungs, myocardium, several lymph nodes, skeletal muscles, and small and large intestines (Figure 3). Furthermore, myofibromas were found even within the leptomeninges of the cerebrum and cerebellum, which has been seldom reported [12]. As per our knowledge, such uncommon and extensive involvement of the lung and leptomeninges has not been previously described.

The histogenesis of neoplastic cells in IM is assumed to be of myofibroblastic lineage with additional features of modified myopericytes [13]. The differential diagnosis includes a variety of mostly benign tumours with similar morphology, namely myopericytoma, leiomyoma, glomus tumour, and solitary fibrous tumour, and in cases of myofibromas with atypical features also infantile fibrosarcoma, leiomyosarcoma, or any other sarcomas with myopericytic characteristics [2]. In diagnostically challenging cases, ancillary techniques (immunohistochemistry, in certain cases also molecular genetic testing) may be of help. In our case, histopathologic findings showed a biphasic growth pattern with a densely cellular central region of immature-looking round cells, surrounded by spindle cells, without cytologic atypia or prominent mitotic activity, which is consistent with findings reported by other authors [3,4]. Immunohistochemically, the spindle myoid cells were positive for smooth muscle actin while negative for desmin, whereas the round cell component showed positivity solely for CD34, which has been described previously [14]. It is noteworthy that atypical histopathological features do not correlate with adverse outcomes [2].

Radiologic findings of IM are not specific. The vast majority of IM with only lung’s involvement present as a solitary nodule of various sizes on radiography [15,16]. The case presented here is unique in its extensive involvement of the lung, resembling a metastatic disease on chest radiograph. Ultrasonography of the lung showed multiple round hypoechoic solid nodules within the lung parenchyma, which were hypovascular on Doppler examination. Although this appearance is not specific for IM, the nodules were similar in appearance to those previously described by other authors [17]. Ultrasonography can be a useful technique even for prenatal detection of the tumours; however, to the best of our knowledge, only scant reports exist on prenatally diagnosed IM with ultrasonography [18]. In our case, the routine prenatal US in the first and second trimester showed no abnormalities. Due to the also unremarkable clinical surveillance of pregnancy, a follow-up prenatal US was not scheduled. It is, however, remarkable that the majority of prenatal cases have been discovered later in the third trimester [18]. Despite the autopsy findings of myofibromas in several visceral organs (i.e., heart, small and large intestine), they were not visualized by the US examinations. It is also noteworthy that, at the time of the examinations, the diagnosis of generalised IM has not been yet settled upon; therefore, the examinations were not specifically performed to search for minor tumours in all various locations [18]. Bones are also often involved in multicentric IM with the skull, tibia, femur, spine, and ribs being the most common locations, usually appearing on radiographic images as well-defined areas with or without sclerotic rings [1,19]. However, there were not any osteolytic lesions observed on radiographic images in our case, nor on autopsy. As aforesaid, myofibromas of bones and soft tissues exhibit variable appearance on conventional radiography and non-specific heterogenous signal density or intensity on CT and MRI as well; therefore, using imaging modalities, per se, cannot confirm the final diagnosis of IM. Nonetheless, some of their MRI characteristics (irregular strip, patchy hypointensities, pseudocapsule at the periphery) might be useful for differentiating them from other soft tissue tumours, although final diagnosis is still made with the histopathological assessment [3,19].

As IM without visceral involvement tends to spontaneously regress, treatment is usually not recommended [1]. In IM with visceral involvement, the most common treatment regimens are an intravenous application of vinblastine and methotrexate; however, some authors recommend interferon α, other chemotherapeutical agents (vincristine, actinomycin D, cyclophosphamide), radiotherapy, and surgical treatment as well [4,20]. In our case, multiple lesions in the lungs prevented adequate ventilation and tissue oxygenation, which led to a progressively deteriorated clinical condition, multiorgan failure, and death, before the patient could receive targeted treatment for IM.

## 4. Conclusions

In conclusion, we presented a unique case of IM with extensive involvement of skin and visceral organs, including the lung, which resembled a metastatic disease on chest radiography. Radiological findings of IM are non-specific, although important in the determination of the extent of disease. IM should always be considered as a differential diagnosis when evaluating a new-born with multiple solid nodules involving the lung or other visceral organs in association with bluish papular skin nodules. As the differential diagnosis of soft tissue tumours is broad, a biopsy of the skin nodules should be performed quickly to reach the correct diagnosis.

## Data Availability

The data supporting the findings of this study are private due to the protection of personal data.
